# Resources and Environment Carrying Capacity, Social Development and Their Decoupling Relationship: A Case Study of Hubei Province, China

**DOI:** 10.3390/ijerph182312312

**Published:** 2021-11-23

**Authors:** Sheng Ye, Chao Wei, Zhanqi Wang, Han Wang, Ji Chai

**Affiliations:** 1 School of Public Administration, China University of Geosciences, Wuhan 430074, China; yesheng@cug.edu.cn (S.Y.); chaiji901027@cug.edu.cn (J.C.); 2 School of Public Administration, Hubei University, Wuhan 430062, China; weichao@hubu.edu.cn; 3 Key Laboratory for Rule of Law Research, Ministry of Natural Resources, Wuhan 430074, China; 4 Key Laboratory for Urban Habitat Environmental Science and Technology, School of Urban Planning and Design, Peking University, Shenzhen 518055, China; han.wang@stu.pku.edu.cn; 5 Key Laboratory for Earth Surface Processes, Ministry of Education, College of Urban and Environmental Sciences, Peking University, Beijing 100871, China

**Keywords:** resources and environment carrying capacity, social comprehensive development index, decoupling model, urban sustainable development, Hubei

## Abstract

With the rapid urbanization in recent decades, resource shortage and environmental damage have hindered the process of urban sustainable development (SD). As a yardstick of sustainable development, the evaluation of resources and environment carrying capacity (RECC) and its decoupling relationship with social comprehensive development index (SCDI) are of great significance. In this paper, RECC and SCDI are taken as research objects to establish resource and environment system evaluation index system and social comprehensive development level evaluation index system, respectively. Then, the RECC and SCDI of 17 cities in Hubei province during 2009–2018 are calculated by the projection pursuit model based on genetic algorithm, and their spatial-temporal variance characteristics are analyzed. On this basis, the RECC-SCDI Tapio decoupling model is constructed to explore the decoupling relationship between RECC and SCDI. The result shows that: (1) The RECC of Hubei shows a V-shaped development trend during 2009–2018. The SCDI of Hubei rose steadily during 2009–2018. (2) RECC in western and eastern Hubei Province is higher than that in central Hubei Province. SCDI in eastern and central Hubei Province is higher than that in the west. (3) 11 of the 17 cities in Hubei Province have got rid of excessive dependence on resources environment for social development. The study could contribute to scientific and effective policies be formulated by government to promote urban sustainable development.

## 1. Introduction

Since the reform and opening up in 1978, China has achieved rapid development and remarkable achievements with the continuous promotion of new urbanization, urban and rural integrated development and rural revitalization strategy [1,2]. However, the consequent accelerated growth of resource consumption and ecological environment deterioration have led to a series of problems, mainly manifested as the intensification of the conflict between production-ecological-living space [3], the degradation of ecosystem functions [4], and the threat to food security [5]. These problems have intensified the regional human–land contradiction and restricted the urban sustainable development seriously [6,7]. How to coordinate the relationship between social and economic development under the constraints on resources and environment to achieve urban sustainable and high-quality development has become a hot issue concerned by the government and scholars of all circles [8,9]. At the end of the 20th century, population, resources, environment and development (PRED), which is the study of man–nature relation has gradually become an important proposition with the emergence of global resource depletion and environmental deterioration [10]. As an important criterion to measure the coordinated development of man–nature relations in a country or region [11], RECC has attracted more and more attention and was introduced to help address resources environment problems in the process of industrialization and urbanization.

RECC is the general name of resources carrying capacity and environmental carrying capacity [8,12]. It may be understood as the maximum amount of resources that nature can provide for human activities and sustaining social development without causing irreversible damage to the ecosystem at the current level of science and technology [13,14,15]. Social development will inevitably consume resources and affect the environment. If the society develops too fast, the carrying capacity of resources and environment will face the risk of overload, and once the carrying capacity of resources and environment is overloaded, the resource–environment–society system will collapse [16]. Therefore, the objective and accurate evaluation of regional RECC and the exploration of its coupling state with social development can provide scientific and effective reference for the formulation of urban development strategies, which is of great significance to promote the sustainable development of urban resources, environment and society [17]. Earlier studies have already raised questions related to RECC and carried out corresponding research work. The study <Limits of growth> [18] published by the Rome Club in 1972 constructed a well-known ‘world model’ by using the system dynamics model to explore regional carrying capacity. They pointed out that if human society continues developing in an uncontrolled manner, regardless of environmental damage, society will collapse within a hundred years. UNESCO and FAO used the Evolution of Capital Creation Options (ECCO) model to estimate the resource carrying capacity (RCC) of Kenya, Mauritius, Zambia and other developing countries [19]. Arrow published <Economic Growth, Carrying Capacity and Environment>, which attracted scholars’ attention to environmental carrying capacity (ECC) [20]. Recently published works showed that scholars have developed wealthy methods and models to carry out a resource and environment assessment. Analytic hierarchy process (AHP) combined with system dynamics (SD) models [21] and cloud model with Markov chain [22] helped to evaluate water resources carrying capacity and atmospheric environmental carrying capacity. GIS-based methodology [23] and the TOPSIS model based on the entropy weight method [24] were generally used in the provincial RECC evaluation works. Coupling coordination degree model (CCDM) [25] could measure whether and to what extent that urbanization growth and RECC were coordinated, thus help to explore relationship between ecological environment carrying capacity and social-economic development. It is not difficult to see from the development context of carrying capacity that carrying capacity research started from the exploration of population growth limit. Subsequently, it was introduced into the field of ecology to study the maximum number of species that a certain region can accommodate. Due to the aggravation of resource shortage and environmental deterioration, the coordination and balance between resource environment and social development as well as the issue of sustainable development have begun to be focused on. The research of carrying capacity has developed from single land resources [26] and water resources carrying capacity [27] to comprehensive research of ecology, resources and environment carrying capacity [25].

**Figure 1:**
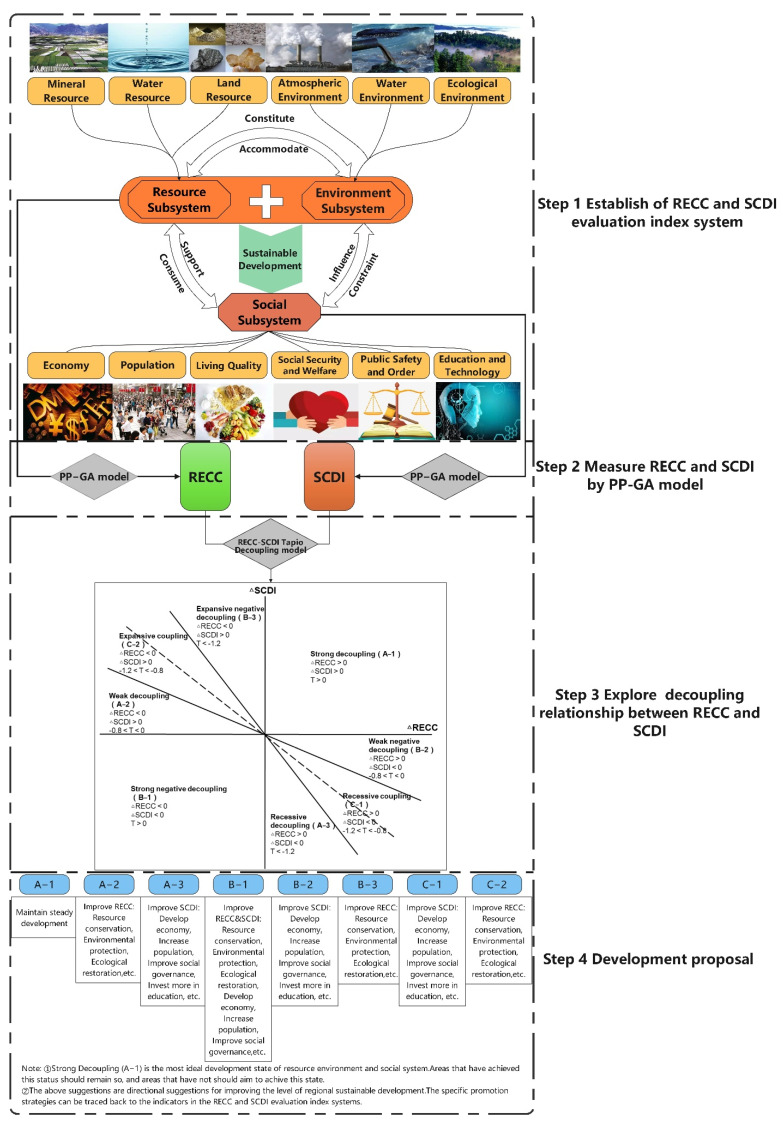
Research framework.

Previous studies about RECC focused on the measurement method and the evaluation of carrying capacity, while there is still less attention to explore the decoupling relationship between RECC and social development [28,29]. What is the decoupling relationship between regional RECC and social development remains unclear. Even in studies exploring the relationship between RECC and social development, social development is often measured by economic indicators or urbanization level [29,30]. However, social development is not just economic growth or the increase in the proportion of urbanization, but the comprehensive development of economy, population, quality of life, social order, public safety, education and science and technology [31,32,33]. To address the above research gaps, we constructed a research framework to evaluate regional RECC, SCDI and to explore the decoupling relationship between them (Figure 1). In the research framework, RECC stands for “Nature” and SCDI stands for “Man”. The RECC-SCDI system is composed of the resource subsystem, the environment subsystem, and the social subsystem. The interconnections between these three subsystems are as follows: Firstly, the resource subsystem, which consists of land resources [34,35,36], water resources [21,37] and mineral resources [29] supports social subsystem by providing food, water, building materials, industrial materials, etc. Social subsystem consumes resources subsystem in return. Secondly, social subsystem influences environment subsystem in both positive and negative ways. For example, the discharge of urban waste gas and wastewater has a negative impact on the environmental subsystem, while the ecological restoration project has a positive impact on the environmental subsystem. In turn, environment subsystem that consists of water environment [38,39,40], atmospheric environment [22,41], and ecological environment [42,43] constraints social subsystem. That is, if the negative impact of social development on the environment exceeds a certain limit, social development will be inhibited. For instance, excessive use of chemical fertilizers in agricultural production will lead to soil contamination, and yield of agricultural products decreases when soil is contaminated. Thirdly, the resources in the resource subsystem constitute the environment subsystem, which in turn accommodates the resources in the resource subsystem. For example, water, animals, and microorganisms in the water together constitute the aquatic environment, which also provides habitat for aquatic organisms. The proper functioning and dynamic balance of each subsystem is the key to the sustainable development of the region.

The relationship between RECC and SCDI may be employed as an “invisible indicator” to judge whether regional development is sustainable. Imbalances on either system can lead to unsustainable development. Decoupling describes the process of diminishing interaction between two systems [44]. Later, it is often applied to the study of relationship between traffic and carbon emissions [45,46], environmental pollution and economic growth [46]. Here, the RECC-SCDI Tapio decoupling model is constructed to evaluate the decoupling stage of RECC and SCDI by calculating the decoupling index. Based on the above understanding, the steps of the research framework are as follows: First, the evaluation index system of RECC and SCDI is constructed, in which RECC consists of 18 indicators in six aspects, and SCDI consists of 21 indicators in six aspects. This index system can provide reference for the evaluation of resources environment and social development in similar non-marine areas. Secondly, in order to avoid the influence of human subjectivity on the evaluation results, the projection pursuit model based on genetic algorithm, which has advantages in processing high-dimensional and nonlinear data is applied to measure RECC and SCDI. Thirdly, the RECC-SCDI Tapio decoupling model is constructed to evaluate the decoupling stage of RECC and SCDI by calculating the decoupling index. Finally, the factors limiting regional sustainable development could be analyzed according to the decoupling index. Thus, corresponding measures and suggestions could be provided to adjust factors in RECC or SCDI in order to promote regional sustainable development.

## 2. Materials and Methods

### 2.1. Study Area

**Figure 2:**
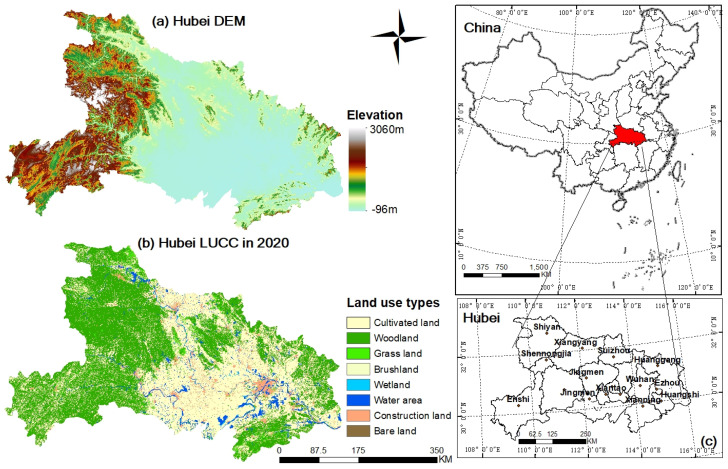
(**a**) The Digital Elevation Model (DEM) of Hubei; (**b**) The Land-Use and Land-Cover Change (LUCC) of Hubei in 2020. (**c**) Location of the study area.

Hubei Province is located in central China, with 17 prefecture-level cities under its jurisdiction, ranging from 29°01′53″~33°6′47″ N latitude and 108°21′42″~116°07′50″ E longitude. The location is shown in Figure 2. Hubei covers an area of 185,900 square kilometers, of which mountains, hills and plains and lakes account for 56%, 24%, and 20%, respectively, and is a typical subtropical monsoon climate type. As an important node province of the Yangtze River Economic Belt (The Yangtze River Economic Belt is one of China’s three key strategies. It covers 11 provinces and cities, covering an area of about 2.05 million square kilometers, accounting for 21.4 percent of China’s land area and accounting for more than 40 percent of the country’s population and GDP), Hubei province shoulders the dual pressure of economic development and ecological civilization construction with the proposal of the development concept of jointly focusing on greater protection and avoiding large-scale development. The permanent resident population of Hubei exceeded 57.75 million in 2020, with an average annual growth rate of 0.09%. The per capita GDP was RMB 75,200 yuan, making Hubei rank first among the six Chinese central provinces (China’s economic regions are divided into eastern, central, western and northeastern regions. The six central provinces include Shanxi, Anhui, Jiangxi, Henan, Hubei and Hunan provinces) and a position of the important strategic fulcrum of regional development. In this context, it is of great reference significance to explore the decoupling path between resources environment, and social development in Hubei Province to alleviate the pressure of economic construction and ecological civilization.

### 2.2. Data Sources

LUCC data were obtained from GlobeLand30 (http://www.globallandcover.com accessed on 2 June 2021), with a resolution of 30 m and a date of 2020. DEM data was derived from the Resource and Environment Data Cloud Platform (https://www.resdc.cn/ accessed on 5 September 2021). Land resources data were collected from the Hubei Provincial Land and Resources Bulletin. Water resources data were collected from the Hubei Water Resources Bulletin. Mineral resources data were from the China Urban Construction Statistical Yearbook. Environmental data came from the Environmental Quality Qtatus Report on Hubei Province and the Environmental Air Quality Report on key cities. Social and economic data were obtained from the Hubei Statistical Yearbook.

### 2.3. Index System

#### 2.3.1. RECC Evaluation Indicators System

Resources and environment are two different subsystems. The various resources in the resource subsystem constitute the environment subsystem. The environment subsystem accommodates the various resources in the resource subsystem. For nearly half a century, the research on natural resources carrying capacity mainly focuses on land resources, water resources and mineral resources [16,47,48], and the environmental carrying capacity mainly focuses on water environmental carrying capacity and ecological environmental carrying [49,50,51]. Considering that in recent years, atmospheric pollution problems such as excessive PM 10 content and excessive greenhouse gas emissions have become increasingly serious [52], this study added atmospheric environmental indicators into the environmental subsystem. Combined with expert consultation and literature review, a total of 18 indicators were selected to construct the evaluation index system of resource and environmental carrying capacity based on the principles of comprehensiveness, scientificalness and data availability (Table 1).

#### 2.3.2. SCDI Evaluation Indicators System

We sorted out the methods and indicators of social development assessment in DECD, UNDP, ASHA (American Social Health Association), China National Bureau of Statistics and other institutions (Table 2). With this as a reference, 21 indexes were selected from the 6 aspects of Economy, Population, Living quality, Social security and welfare, Public safety and order, Education and technology to construct the evaluation index system of social comprehensive development index (Table 3).

### 2.4. Construction of Data Matrix

Form the initial data matrix of the evaluation system:

(1) $$x_{ij}=\left(\begin{array}{ccc} x_{11} & \cdots & x_{1n}\\ \vdots & \ddots & \vdots \\ x_{m1} & \cdots & x_{mn} \end{array}\right)$$

where,$\;x_{ij}$ represent the value of the *i*th indicator in the *j*th sample. (0 ≤ *i* ≤ *m*, 0 ≤ *j* ≤ *n*).

### 2.5. Standardization of Indicators

Before the evaluation, the evaluation indicators should be standardized in order to eliminate the influence of different dimensions on the evaluation results. Considering the promoting and inhibiting effects of positive and negative indicators on the evaluation results, the extreme value method is adopted to standardize the evaluation indexes, and the formulas are as follows:

Positive indicators:

(2) $${x^{\prime }}_{ij}=\frac{x_{ij}-x_{min}}{x_{max}-x_{min}},\;(0\;\leq \;i\;\leq \;m,\;0\;\leq \;j\;\leq \;n)\;$$

Negative indicators:

(3) $${x^{\prime }}_{ij}=\frac{x_{max}-x_{ij}}{x_{max}-x_{min}},\;(0\;\leq \;i\;\leq \;m,\;0\;\leq \;j\;\leq \;n)\;$$

where, ${x^{\prime }}_{ij}$ is a normalized indicator;$\;x_{ij}$ is pre-normalized indicator; $x_{max}$ is the maximum value among column *j*; $x_{min}$ is the minimum value among column *j*.

### 2.6. Projection Pursuit Model Based on Genetic Algorithm

The projection pursuit model is a multi-dimensional data processing method that projects high-dimensional data to one-dimensional space and retains the most data information by finding the optimal projection direction [57]. Compared with traditional evaluation methods, such as the analytic hierarchy process and the Delphi method, the projection pursuit model has the advantage of avoiding artificial subjective factors and processing nonlinear distributed data [58,59,60]. Here, projection pursuit model based on a genetic algorithm is used to evaluate the RECC and SCDI. The modeling steps are as follows:

#### 2.6.1. Construction of Projection Indicator Function

Set $v_{j}=\left\{v\left(1\right),v\left(2\right),v\left(3\right),\ldots ,v\left(j\right)\right\}$ as the projection direction vector of each index in the evaluation of resource and environment carrying capacity or social development level, then the one-dimensional projection value of the *i*th sample in the projection direction can be calculated by the following formula:

(4) $$Z\left(i\right)=\overset{n}{\underset{j=1}{\sum }}v_{j}\times {x^{\prime }}_{ij}\;\left(i=1,2,3,\ldots ,m\right)$$

where, Z(i) is sample projection value; $v_{j}$ is projection direction vector; ${x^{\prime }}_{ij}$ is the normalized indicator.

#### 2.6.2. Construction of Projection Objective Function

For the projection direction vectors in the above steps, different projection directions can reflect different data information. In order to make the evaluation results cover all data information, the objective function is constructed as follows:

(5) $$Q\left(v\right)=S_{z}\times D_{z}$$

(6) QSz =∑i=1m(Z(i)−E(z))2n−1

(7) $$D_{z\;}=\overset{m}{\underset{i=1}{\sum }}\overset{n}{\underset{j=1}{\sum }}\left(R-r\left(i,j\right)\right)\times f(R-r\left(i,j\right)$$

(8) r(i,j)=|Z(i)−Z(j)|

(9) $$\{\begin{array}{c} f(R-r\left(i,j\right)=1,R>r\left(i,j\right)\\ f(R-r\left(i,j\right)=0,R\leq r\left(i,j\right) \end{array}$$

where, Q(v) is projection objective function; $S_{z\;}$ is the standard deviation of Z(i); $D_{z}$ is the local density of Z(i); E(z) is the average value of Z(i); *R* is the window radius of the local density, which is related to the data characteristics; r(i,j) is the distance between the samples; f(m) is the unit step function.

#### 2.6.3. Optimization of Projection Objective Function by Genetic Algorithm

Different projection direction reflect different data structure. To calculate the optimal projection direction, the projection objective function must be maximized:

(10) $$Max:Q\left(v\right)=S_{z}\times D_{z}$$

The constraint condition is:

(11) $$\overset{n}{\underset{j=1}{\sum }}a^{2}\left(j\right)=1\;$$

To solve the complex nonlinear optimization variable problem, this paper uses Genetic Algorithm to search for global optimization, and obtains the best projection direction vector$\;v_{j}{}^{*}$. This process is achieved by software programming in MATLAB.

#### 2.6.4. Calculation of Projection Value

After the optimal projection vector is calculated, the projection value of each sample can be calculated by substituting the optimal projection vector $\;v_{j}{}^{*}$ into Formula (4). The projection value of each sample is the evaluation result of each sample.

### 2.7. RECC-SCDI Tapio Decoupling Model

The RECC-SCDI Tapio decoupling model was constructed to evaluate decoupling stage of RECC and SCDI. The formula is as follows:

T = (%ΔRECC/RECC)/(%ΔSCDI/SCDI) (12)

where, T is the decoupling index between RECC and SCDI; RECC is Resource and Environment Carrying Capacity; %ΔRECC is the percentage change of Resource and Environment Carrying Capacity; SCDI is Social Comprehensive Development Index; %ΔSCDI is the percentage change of Social Comprehensive Development Index. After calculating the decoupling index, the decoupling state can be judged by rules of decoupling state judgment between RECC and SCDI (Table 4). In the table, A-1 represents the strong decoupling state, indicating that RECC and SCDI are rising simultaneously, which is the optimal sustainable development state. A-2 represents a weak decoupling state, indicating that with the increase of SCDI, RECC is decreasing, while the elastic coefficient T is between −0.8 and 0, indicating that the increase of SCDI does not depend too much on the decrease of RECC. A-3 represents Recessive decoupling, indicating that SCDI is decreasing with the increase of RECC and the elastic coefficient T is less than −1.2. It shows that the increase of RECC is not too dependent on the decrease of SCDI. B-1 represents a strong negative decoupling state. It means that with the reduction of RECC, SCDI is also decreasing, which is the worst development state. B-2 represents a weak negative decoupling state, indicating that with the increase of RECC, SCDI is decreasing and the elastic coefficient T is between −0.8 and 0, indicating that the increase of RECC does not depend too much on the decrease of SCDI. B-3 represents expansive negative decoupling state, indicating that RECC is decreasing with the increase of SCDI. The elastic coefficient T is less than −1.2, indicating that the increase of SCDI does not depend too much on the decrease of RECC. C-1 represents the state of recessive coupling, indicating that SCDI decreases with an increase of RECC, while the elastic coefficient T is between −1.2 and −0.8, indicating that the increase of RECC is highly dependent on the decrease of SCDI. C-2 represents the expensive coupling state, indicating that RECC is decreasing with the increase of SCDI. The elastic coefficient T is between −1.2 and −0.8, indicating that the increase of SCDI is highly dependent on the decrease of RECC.

## 3. Results

### 3.1. RECC of Hubei Province

#### 3.1.1. Temporal Evolution Characteristics of RECC

**Figure 3:**
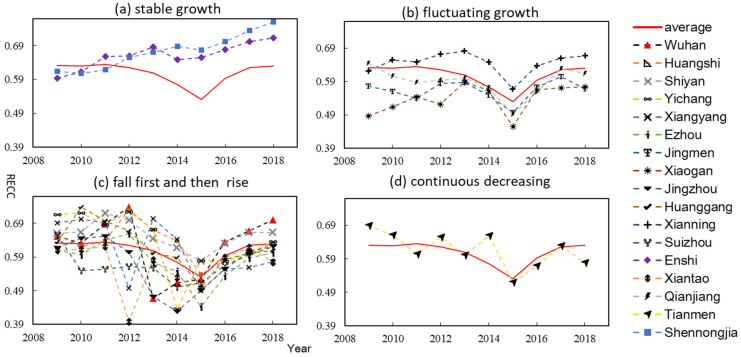
Trend of RECC in Hubei Province during 2009–2018: (**a**) the stable growth of RECC; (**b**) the fluctuating growth of RECC; (**c**) the RECC fall first and then rise; (**d**) the continuous decreasing of RECC.

The time-series results showed the trend of RECC in Hubei Province during 2009–2018. The results are shown in Table A1 in Appendix A. We divided the changes of RECC of cities into four categories (Figure 3. The first category is the stable growth of RECC, and these cities include Shennongjia, Enshi. The second category is fluctuating growth, the overall resource and environmental carrying capacity of these cities is rising, but there is certain volatility in the rising process, including Jingmen, Xiaogan, Xianning and Qianjiang. The third category showed that RECC first declines and then rises, and these cities include Wuhan, Huangshi, Shiyan, Yichang, Xiangyang, Ezhou, Jingzhou, Huanggang, Suizhou and Xiantao. The fourth type is continuous decreasing, including Tianmen. In these cities, the highest RECC was Shennongjia in 2018 (0.7593), and the lowest was Xiantao in 2012 (0.3984), with an average of 0.6084. From the perspective of the evaluation index, the reasons for the high RECC in Shennongjia are its rich forest resources, good atmospheric environment, water environment and ecological environment. The average RECC first decreased and then increased, with a minimum value of 0.5305 in 2015 and a maximum value of 0.63501 in 2011, showing that the level of RECC in Hubei Province gradually improved after 2015.

#### 3.1.2. Spatial Distribution Characteristics of RECC

**Figure 4:**
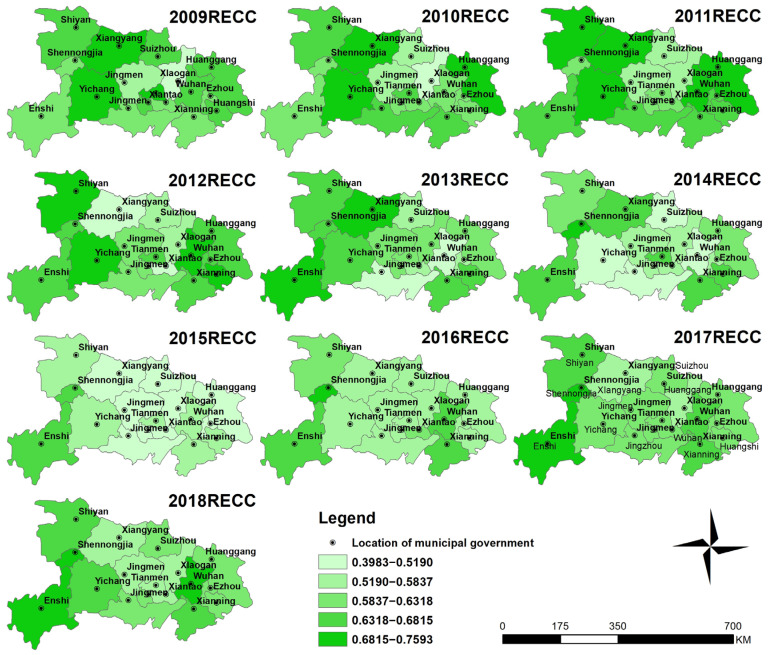
The spatial distribution of RECC in Hubei Province during 2009–2018.

The spatial distribution of RECC levels during 2009–2018 is shown in Figure 4. The natural breaks method was employed to classify the RECC level into five grades, where the first grade indicates the lowest RECC level and the fifth indicates the highest. Overall, RECC in western and eastern Hubei Province was higher than that in central Hubei Province. During 2009–2018, the RECC has continued to grow in the western region except for Shiyan. Among them, Shennongjia had the largest increase, from 0.6143 in 2009 to 0.7593 in 2018, with an increase of 23.6%. In central and western regions, except for Wuhan, Xianning, Jingzhou and Xiaogan, RECC showed an overall downward trend, with Tianmen showing the largest decrease, from 0.6892 in 2009 to 0.5785 in 2018, a decrease of 16.71%.

### 3.2. SCDI of Hubei Province

#### 3.2.1. Temporal Evolution Characteristics of SCDI

**Figure 5:**
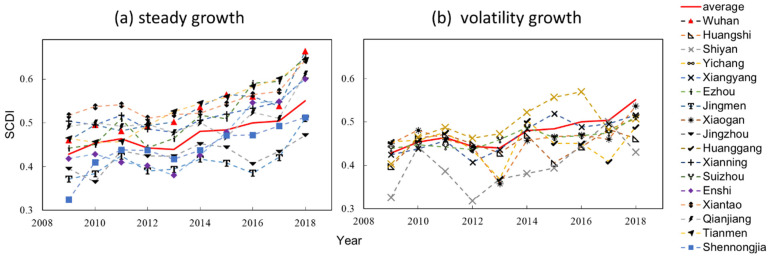
Trend of SCDI in Hubei Province during 2009–2018: (**a**) the steady growth of SCDI; (**b**) the volatility growth of SCDI.

The time-series results show the trend of SCDI in Hubei Province during 2009–2018 (Figure 5). There were two categories in the changes of SCDI. The first type was steady growth, and this category includes Wuhan, Ezhou, Jingmen, Jingzhou, Xianning, Enshi, Xiantao, Qianjiang, Tianmen, Shennongjia. The second type was volatility growth, which includes Huangshi, Shiyan, Yichang, Xiangyang, Xiaogan, Huanggang, Suizhou. The highest SCDI is Wuhan in 2018 (0.6646), and the lowest is Shiyan in 2012 (0.3183), with an average of 0.6084. The average SCDI shows a sustained growth trend, the lowest value is 0.4284 in 2009, and the highest value is 0.5508 in 2018. It shows that the level of social comprehensive development in Hubei Province is steadily improving.

#### 3.2.2. Spatial Distribution Characteristics of SCDI

**Figure 6:**
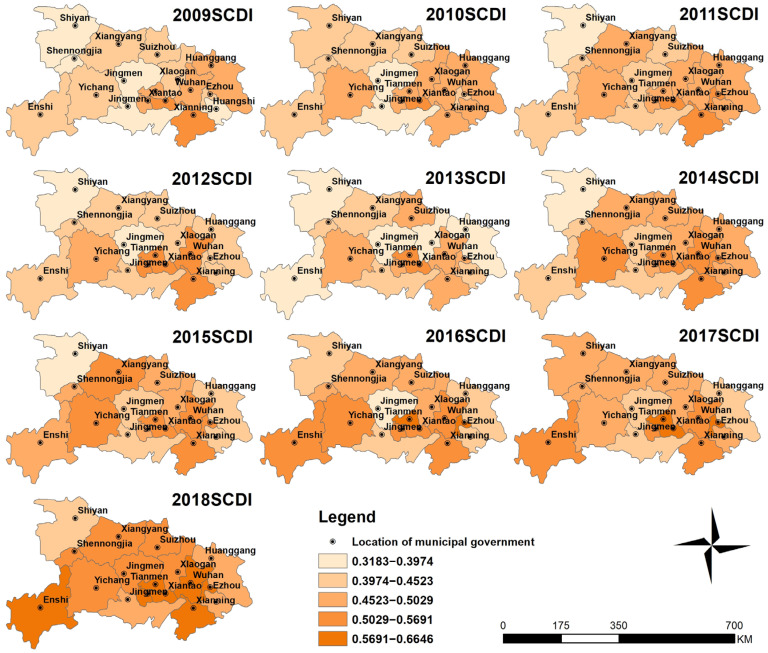
The spatial distribution of SCDI in Hubei Province during 2009–2018.

The spatial distribution of SCDI levels during 2009–2018 is shown in Figure 6. SCDI levels were classified using the same classification method as RECC. Overall, SCDI in eastern and central Hubei Province was higher than that in the western part. During 2009–2018, SCDI of 17 cities in Hubei province grew steadily, with an average increase of 28.57%. The biggest increase was in Shennongjia, which increased 57.86% from 0.3248 in 2009 to 0.5127 in 2018.Huanggang saw the smallest increase, from 0.4547 in 2009 to 0.4885 in 2018, with an increase of 7.44%. It is worth noting that Shennongjia’s SCDI in 2009 was the lowest in Hubei while its growth rate was now the first, indicating that the differences in social and economic development between regions are narrowing.

### 3.3. Decoupling Stage between RECC and SCDI of Hubei Province

**Figure 7:**
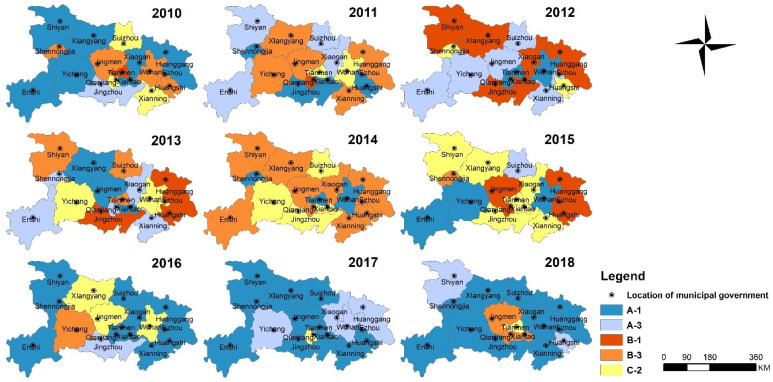
Decoupling states between RECC and SCDI of Hubei Province during 2010–2018.

The spatial distribution of decoupling stages for the period 2010–2018 is shown in Figure 7. The results show that there are five kinds of decoupling states: Strong decoupling (A-1), Recessive decoupling (A-3), Strong negative decoupling (B-1), Expansive negative decoupling (B-1) and Expansive coupling (C-2). On the whole, the decoupling status of cities in Hubei Province fluctuated greatly from 2010 to 2015, and the decoupling status tended to be stable from 2016 to 2018, and 11 cities reached strong decoupling. Among them, there are five decoupling states in 2010, 2011, 2013 and 2015 with large regional differences. 2018 was the most coordinated year for RECC and SCDI, with 5 cities in a state of strong decoupling. While the most incongruous year was 2012, with 6 cities in strong negative decoupling.

**Figure 8:**
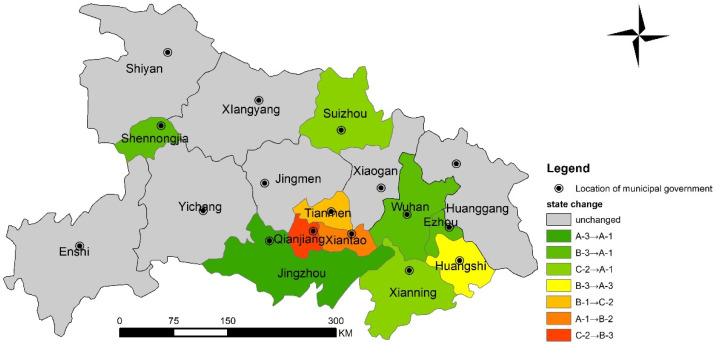
Decoupling states change between RECC and SCDI of Hubei Province during 2010–2018.

The spatial distribution of decoupling stages changes during 2010–2018 is shown in Figure 8. 7 out of the 17 cities showed no change. Two cities (Jingzhou, Shennongjia) changed from A3 to A1. Two cities (Wuhan, Ezhou) changed from B3 to A1. 2 cities (Xiangyang, Suizhou) change from C2 to A1. Huangshi changed from B3 to A3. Tianmen changed from B1 to C2. Xiantao changed from A1 to B2. Qianjiang changed from C3 to B3. The results showed that the decoupling status fluctuates greatly across regions, with a general move toward strong decoupling.

## 4. Discussion

In order to explore the decoupling relationship between resources and environment carrying capacity and social development, this paper established the RECC and SCDI evaluation index system and calculated the RECC and SCDI of Hubei Province during 2009–2018 by the projection pursuit model based on genetic algorithm. Then, the RECC-SCDI Tapio decoupling model was constructed to calculate the decoupling index between RECC and SCDI in Hubei Province during 2010–2018.

From the perspective of temporal evolution characteristics of RECC, the average value of RECC in Hubei Province during 2009–2018 showed a trend of first decreasing and then increasing. Decline to a minimum in 2015 and then rise gradually, eventually formed a ‘V’ curve. This finding may be explained as follows. Hubei Province is located in central China and an important node of the Yangtze River Economic Belt. The Yangtze River Economic Belt, which spans three major regions in China, is one of the ‘three major strategies’ implemented by the central government. It is also an advanced demonstration zone of ecological civilization construction [61]. On 5 January 2016, the symposium on promoting the development of the Yangtze River Economic Belt was held, putting the restoration of the ecological environment of the Yangtze River in an overwhelming position, and paying great attention to protection and development. Affected by this policy, Hubei Province has changed the way of economic development at the cost of excessive consumption on resources and environmental destruction, and has begun to pay attention to resource conservation and utilization, ecological environment protection and restoration. Therefore, in 2016, the utilization of resources was efficiently saved, the ecological environment was restored and protected. Thus the RECC was significantly improved. This shows that regional resources and environment conditions are greatly influenced by policy. This is similar to the existing research that the RECC of Anhui Province presents a “V” shape [24], that of large cities in China presents a “V” shape [8]. The difference between this paper and the existing research is that this paper established the evaluation index system of resources and environment system and social system as two independent systems, instead of combining the two systems into a whole, which can present the regional natural resource endowment and social development more clearly. Then, the decoupling relationship between the two systems has been further analyzed to diagnose whether the two systems are in a sustainable development state and provide scientific suggestions accordingly. From the perspective of spatial distribution characteristics of RECC and SCDI, RECC in western and eastern Hubei Province is higher than that in central Hubei Province and SCDI in eastern and central Hubei Province is higher than that in the west. This finding may be explained as follows. On the one hand, the terrain of Hubei Province is high in the west and low in the east (DEM in Figure 2), and 80% of woodland land is distributed in western and eastern Hubei (LUCC in Figure 2). As is known to all, woodland land is an important type of ecological land, which plays an extremely important ecological function in the ecosystem and can regulate the regional environmental conditions effectively. Therefore, the environmental conditions in the west and east are better than the central Hubei. On the other hand, the Major Function Area Planning of Hubei Province has made a development orientation for all cities of Hubei Province. It is determined in the planning that most of the western cities and some of the eastern cities are positioned as key ecological function areas. The region mainly provides ecological products, and large-scale, high-intensity industrialization and urbanization development in the development of land space are restricted to maintain and improve the supply capacity of ecological products. Cities in the central Hubei are positioned as the main producing areas of agricultural products, which mainly develop agriculture to ensure the safety of national agricultural products supply. Cities in the eastern Hubei represented by Wuhan are positioned as key development zones. This part of the region refers to the region with strong economic foundation, certain scientific and technological innovation ability and good development potential, which can promote the development of surrounding areas and focus on industrialization and urbanization. Therefore, the overall level of social development in the eastern region is higher than that in the central and western regions. The evaluation results of RECC and SCDI are basically consistent with the positioning of each city in the Major Function Area Planning of Hubei Province. Cheng et al. also found that regional resources and environment are influenced by main function zone planning in their evaluation study of RECC in 31 provinces of China in 2016 [17]. In this study, we not only verified this conclusion, but also found that regional socio-economic development was affected by resource and environment endowment and regional development planning.

**Figure A1:**
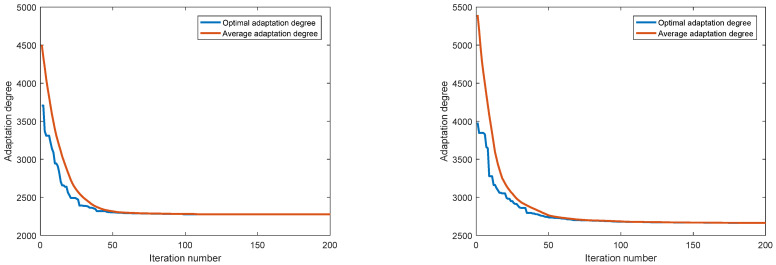
Objective function adaptation degree curve of RECC (**left**) and SCDI (**right**).

From the perspective of decoupling relationship between RECC and SCDI, according to the results of RECC and SCDI decoupling index of cities in Hubei Province in 2018, six cities failed to reach strong decoupling status (A-1), namely Huangshi city (A-3), Shiyan City (A-3), Jingmen City (B-3), Xiantao City (B-3), Qianjiang City (B-3) and Tianmen City (C-2). Huangshi and Shiyan did not achieve strong decoupling because of negative growth of SCDI. Jingmen, Xiantao, Qianjiang and Tianmen city because of negative growth of RECC. In order to provide scientific suggestions for sustainable development between resources, environment and society in these cities, we further analyzed the limiting factors by the following steps: The contribution degree of each indicator to SCDI was calculated by multiplying the standardized value of each indicator in the SCDI indicator system of Huangshi and Shiyan by its weight, and then sorted. The accuracy results are shown in Figure A1 in Appendix A. The indicator with the lowest contribution degree was the SCDI limiting factor. Similarly, the contribution degree of each index to RECC is calculated by multiplying the standardized value of each index in the RECC index system of Jingmen, Xiantao, Qianjiang and Tianmen by its weight, and then sorted. The index with the lowest contribution degree is the RECC limiting factor. According to the above calculation, SCDI limiting factor of Huangshi and Shiyan is added value of high-tech industry. The RECC limiting factors of Jingmen, Xiantao, Qianjiang and Tianmen are grassland area, woodland area, grassland area and biological abundance index, respectively.

This study has several limitations. First, The RECC evaluation index system constructed in this study is only applicable to cities in inland provinces similar to Hubei province. For cities in special areas, the index can be modified or added appropriately. For example, for areas with serious soil pollution, the index of soil pollution can be added. Second, the evaluation of RECC in this study is based on a closed system. However, the actual situation is that there is a large exchange of resources between regions, which has a great impact on RECC. Therefore, in future studies, factors characterizing the intensity of regional resource exchange can be introduced to modify the RECC evaluation system, so as to make the evaluation results of RECC more accurate and more suitable for the actual situation.

## 5. Conclusions

In order to solve the problems that RECC research is not comprehensive in reflecting the social system and there are few studies on the decoupling relationship between resource and environment system and social system, this paper established RECC and SCDI evaluation systems, respectively, to reflect the situation of regional resource and environment system and social system comprehensively and objectively. At the same time, RECC-SCDI Tapio decoupling model was constructed to explore the decoupling relationship between them. An empirical study is conducted in Hubei Province. The main conclusions can be drawn as follows: Firstly, RECC of Hubei Province has gradually improved after experiencing a trough in 2015, eventually formed a ‘V’ curve. SCDI has continued to rise. Secondly, RECC in western and eastern Hubei Province was higher than that in central Hubei Province. SCDI in eastern and central Hubei Province is higher than that in the west. The main reasons for this result are the difference of topography and geomorphology as well as the difference of development strategy in Hubei Province. Thirdly, from the perspective of the decoupling relationship between RECC and SCDI, ten cities reach A-1 (strong decoupling state) in 2018. This indicates that the social development of these cities no longer relies too much on the consumption of resources and the destruction of the environment, and has reached the stage of development that resources, environment and society are jointly promoted. Shiyan and Huangshi are under the state of A-3 (recessive decoupling state), that is, social development lags behind the improvement of the RECC. Jingmen, Xiantao and Qianjiang are under the state of B-3 (expansive negative decoupling state), that is, social development is not free from excessive dependence on resources and the environment. Tianmen is under the state of C-2 (expansive coupling state), shows that social development depends on resources and environment.

Based on the analysis of the decoupling status of all cities in Hubei province in 2018, the following specific suggestions are put forward for the future development of each city. First, Wuhan, Yichang, Xiangyang, Ezhou, Xiaogan, Jingzhou, Huanggang, Suizhou, Enshi, Shennongjia, as these cities have reached a state of strong decoupling of RECC and SCDI, i.e., social development is no longer overly dependent on resource consumption and environmental pollution, and is a sustainable development state. They can continue to maintain the current pace in future development. Second, Huangshi and Shiyan are in a state of recessive decoupling, i.e., resource consumption and environmental pollution have improved, but the level of comprehensive social development has decreased. According to the analysis of limiting factors, the decline of SCDI in Huangshi and Shiyan is mainly due to the added value of high-tech industry, which means that these two cities should increase the investment in high-tech industry in their future development. Third, Jingmen, Xiantao and Qianjiang are in the state of expansive negative decoupling, indicating that the social development of these three cities still excessively depends on resource consumption and environmental pollution. Among them, Jingmen and Qianjiang should pay attention to the protection of grassland resources, and may carry out projects such as returning farmland to grass, while Xiantao should pay attention to the protection of forest resources. Projects such as returning farmland to forest may be carried out to promote the carrying capacity of resources and environment. Finally, Tianmen is in the state of expansive coupling, i.e., the dependence of social development on resources and environmental systems is high, but lower than Jingmen, Xiantao and Qianjiang. Its RECC enhancement limit factor is the biological abundance index. In the future, Tianmen need to pay attention to the management of “ecological red line”, strengthen ecological protection, and improve the biological abundance index.

This study could be used as a reference to evaluate RECC, SCDI and explore the decoupling relationship between them in non-coastal areas like Hubei. According to the decoupling state between RECC and SCDI, local governments may formulate differentiated control policies to reduce the dependence of social development on resource consumption and environmental damage, and finally achieve strong decoupling between resources environment and society and sustainable development.

## Figures and Tables

**Table 1:** Resources and environment carrying capacity (RECC) evaluation indicators.

Factor	Indicator	No.	Unit	Attribute
Land resource	Cultivated area	R1	Hectare	+
	Woodland area	R2	Hectare	+
	Grassland area	R3	Hectare	+
	Construction area	R4	Hectare	+
Water resource	Total water resources	R5	1000 million m^3^	+
	Amount of water supply	R6	1000 million m^3^	+
Mineral resource	Natural gas supply	R7	10,000 m^3^	+
	Energy consumption per unit GDP	R8	tones of standard coal/10,000 CNY	−
	Coal consumption	R9	10,000 t	+
Atmospheric environment	Air quality excellent rate	R10	%	+
	Annual average PM10 concentration	R11	mg/m^3^	−
	Air quality composite index	R12	/	−
Water environment	Industrial wastewater effluent	R13	10,000 t	−
	Centralized treatment rate of urban sewage	R14	%	+
	City daily sewage treatment capacity	R15	10,000 t	+
Ecological environment	Biological abundance index	R16	/	+
	Vegetation coverage index	R17	/	+
	Water network density index	R18	/	+

Note: “+”means a positive indicator, “−”means a negative indicator.

**Table 2:** Evaluation indicators of social development level in different countries/organizations.

Institution	Document	Index/Model	Indicators (Example)
DECD	The OECD List of Social Indicators [53]	social background; self-sufficiency; fairness; health conditions; social cohesion.	Per capita national income, birth rate, divorce rate; Rate of unemployment, level of education, Basic subsidies; Public social expenditure, level of pensions; Average life, total health expenditure; Subjective well-being, suicide rate.
UNDP	Human development report 2020 [54]	HDI (Human Development Index)	Income; Education; Health; Gender development index; Poverty index.
ASHA (American Social Health Association)	ASHA Index Model [55]	ASHA Index	Rate of employment; Rate of literacy; Average life; Per capita gross national product; Birth rate; Infant mortality rate.
China National Bureau of Statistics	Comprehensive evaluation scheme of social development level [56]	population development; living standard; public services; social harmony.	Population growth rate, average life expectancy; Engel coefficient, disposable income of urban residents, and per capita housing area; Incidence of infectious diseases, basic social insurance coverage, number of beds in welfare units; Urban registered unemployment rate, number of criminal cases, urban-rural income ratio.

**Table 3:** Social comprehensive development index (SCDI) evaluation indicators.

Factor	Indicator	No.	Unit	Attribute
Economy	GDP	S1	10,000 million CNY	+
	GDP growth rate	S2	%	+
	Tertiary industries as a percentage of GDP	S3	%	+
	Fixed asset investment	S4	10,000 million CNY	+
Population	Number of resident population	S5	10,000 person	+
	Population density	S6	Person/hectare	+
Living quality	Urban per capita disposable income	S7	Yuan	+
	Rural net income per capita	S8	Yuan	+
	Urban per capita housing construction area	S9	Person/m^2^	+
	The green coverage rate of the built district	S10	%	+
	Number of Internet users	S11	10,000 households	+
Social security and welfare	Expenditure for general public service	S12	10,000 yuan	+
	Number of social insurance participants	S13	10,000 Person	+
	Registered urban unemployment rate	S14	%	−
	Number of beds in health services	S15	10,000 beds	+
Public safety and order	Number of criminal cases filed	S16	/	−
	Number of the death in traffic accidents and fire	S17	Person	−
Education and technology	Number of full-time teachers	S18	10,000 Person	+
	Number of students in colleges and universities	S19	10,000 person	+
	Patent number	S20	/	+
	Value-added of high-tech industries	S21	1000 million CNY	+

Note: “+”means a positive indicator, “−”means a negative indicator.

**Table 4:** Rules of decoupling state judgment between RECC and SCDI.

Decoupling State		ΔRECC	ΔSCDI	T	Code
Decoupling	Strong decoupling	>0	>0	>0	A-1
	Weak decoupling	<0	>0	−0.8 < t < 0	A-2
	Recessive decoupling	>0	<0	<−1.2	A-3
Negative decoupling	Strong negative decoupling	<0	<0	>0	B-1
	Weak negative decoupling	>0	<0	−0.8 < t < 0	B-2
	Expansive negative decoupling	<0	>0	<−1.2	B-3
Coupling	Recessive coupling	>0	<0	−1.2 < t < −0.8	C-1
	Expansive coupling	<0	>0	−1.2 < t < −0.8	C-2

## Data Availability

Not applicable.

## Appendix A

When using programming language to complete genetic algorithm in MATLAB, this paper set Maximum Iterations = 200, Population Size = 200, Mutation Rate = 0.2, Crossover Rate = 0.85, Catastrophe Rate = 0.1. The adaptive curves of the objective function of RECC and SCDI tend to be stable (Figure A1), indicating that the genetic algorithm has a good effect.

The best projection direction vector${\;v}_{j-\mathrm{RECC}}{}^{*}\;$= (0.02263, 0.00129, 0.00146, 0.00192, 0.00234, 0.00551, 0.11720, 0.00138, 0.03398, 0.20660, 0.20928, 0.15728, 0.12246, 0.00427, 0.00191, 0.00146, 0.05092, 0.05812). The best projection direction vector${\;v}_{j-\mathrm{SCDI}}{}^{*}$ = (0.00079, 0.28607, 0.05454, 0.00200, 0.00121, 0.00199, 0.19907, 0.00658, 0.15592, 0.09895, 0.00233, 0.00103, 0.00041, 0.11640, 0.00220, 0.00228, 0.06192, 0.00067, 0.00079, 0.00449, 0.00036)

**Table A1:** RECC, SCDI and decoupling index of cities in Hubei during 2009–2018.

Cities	Year	RECC	SCDI	Tapio Index	Decoupling State
Wuhan	2009	0.6512	0.4600	-	-
	2010	0.6318	0.4955	−0.3033	B-3
	2011	0.6883	0.4817	−2.2462	C-2
	2012	0.7381	0.4935	1.9835	A-1
	2013	0.4667	0.5021	−22.5700	C-2
	2014	0.5129	0.5363	1.5172	A-1
	2015	0.5238	0.5652	0.4272	A-1
	2016	0.6340	0.5605	−22.3052	C-2
	2017	0.6680	0.5385	−1.1028	A-3
	2018	0.7011	0.6646	0.2003	A-1
Huangshi	2009	0.6549	0.3974	-	-
	2010	0.6214	0.4628	−0.2313	B-3
	2011	0.6386	0.4707	1.1788	A-1
	2012	0.7311	0.4501	−2.0308	C-2
	2013	0.6706	0.4285	1.1056	B-1
	2014	0.6377	0.4695	−0.3768	B-3
	2015	0.5377	0.4054	0.8657	B-1
	2016	0.5802	0.4425	0.6592	A-1
	2017	0.5989	0.4828	0.2845	A-1
	2018	0.6312	0.4612	−0.8826	A-3
Shiyan	2009	0.6613	0.3255	-	-
	2010	0.6643	0.4420	0.0085	A-1
	2011	0.7210	0.3860	−0.3606	A-3
	2012	0.6999	0.3183	0.0760	B-1
	2013	0.6456	0.3685	−0.2807	B-3
	2014	0.6177	0.3809	−0.7912	B-3
	2015	0.5780	0.3937	−1.3015	C-2
	2016	0.6302	0.4463	0.4797	A-1
	2017	0.6631	0.5005	0.3237	A-1
	2018	0.6632	0.4299	−0.0006	A-3
Yichang	2009	0.7162	0.4031	-	-
	2010	0.7211	0.4652	0.0287	A-1
	2011	0.6859	0.4867	−0.7490	B-3
	2012	0.7242	0.4624	−0.7143	A-3
	2013	0.6728	0.4723	−2.3232	C-2
	2014	0.4357	0.5223	−3.9930	C-2
	2015	0.5794	0.5569	4.7897	A-1
	2016	0.5782	0.5691	−0.0952	B-3
	2017	0.6014	0.4821	−0.2108	A-3
	2018	0.6356	0.5074	0.8617	A-1
Xiangyang	2009	0.6910	0.4245	-	-
	2010	0.7020	0.4380	0.3127	A-1
	2011	0.6933	0.4566	−0.1913	B-3
	2012	0.4977	0.4073	2.1356	B-1
	2013	0.7046	0.4361	3.6380	A-1
	2014	0.6428	0.4841	−0.5995	B-3
	2015	0.4900	0.5192	−3.4693	C-2
	2016	0.5539	0.4879	−1.9034	C-2
	2017	0.5584	0.4953	0.4778	A-1
	2018	0.5755	0.5156	0.6719	A-1
Ezhou	2009	0.6107	0.4420	-	-
	2010	0.5967	0.4505	−0.9029	B-3
	2011	0.6410	0.5094	0.4496	A-1
	2012	0.6592	0.4432	−0.1473	A-3
	2013	0.5736	0.4638	−2.2655	C-2
	2014	0.5486	0.5175	−0.3543	B-3
	2015	0.4429	0.5099	15.0296	B-1
	2016	0.5341	0.5908	1.4361	A-1
	2017	0.5875	0.5951	13.9362	A-1
	2018	0.6030	0.6500	0.3092	A-1
Jingmen	2009	0.5762	0.3717	-	-
	2010	0.5608	0.3840	−0.5531	B-3
	2011	0.5388	0.4244	−0.2931	B-3
	2012	0.5837	0.3903	−0.6922	A-3
	2013	0.5865	0.3940	0.3327	A-1
	2014	0.5488	0.4173	−0.8255	B-3
	2015	0.4968	0.4085	3.6918	B-1
	2016	0.5731	0.3851	−1.7990	C-2
	2017	0.6039	0.4212	0.4013	A-1
	2018	0.5687	0.5104	−0.2475	B-3
Xiaogan	2009	0.4864	0.4501	-	-
	2010	0.5148	0.4810	0.7959	A-1
	2011	0.5438	0.4598	−1.0770	A-3
	2012	0.5226	0.4430	0.9041	B-1
	2013	0.5895	0.3581	−0.4058	A-3
	2014	0.5631	0.4573	−0.1310	B-3
	2015	0.4556	0.4658	−10.5187	C-2
	2016	0.5653	0.4696	24.4218	A-1
	2017	0.5705	0.4603	−0.3781	A-3
	2018	0.5746	0.5365	0.0396	A-1
Jingzhou	2009	0.6168	0.3952	-	-
	2010	0.6449	0.3650	−0.3371	A-3
	2011	0.6526	0.4366	0.0404	A-1
	2012	0.6054	0.4244	1.8208	B-1
	2013	0.4727	0.4241	249.7213	B-1
	2014	0.4274	0.4523	−1.5226	C-2
	2015	0.4916	0.4441	−7.4873	C-2
	2016	0.5541	0.4066	−1.1012	A-3
	2017	0.6065	0.4343	0.9923	A-1
	2018	0.6227	0.4718	0.2352	A-1
Huanggang	2009	0.6353	0.4547	-	-
	2010	0.7394	0.4578	14.9250	A-1
	2011	0.6977	0.4756	−0.9864	B-3
	2012	0.6682	0.4354	0.3263	B-1
	2013	0.6023	0.3684	0.3916	B-1
	2014	0.5897	0.5029	−0.0488	B-3
	2015	0.5016	0.4507	1.2919	B-1
	2016	0.5828	0.4509	288.4074	A-1
	2017	0.6011	0.4089	−0.2292	A-3
	2018	0.6089	0.4885	0.0535	A-1
Xianning	2009	0.6218	0.5038	-	-
	2010	0.6547	0.4956	−2.4606	C-2
	2011	0.6489	0.5176	−0.1597	B-3
	2012	0.6717	0.4858	−0.4155	A-3
	2013	0.6815	0.4785	−0.6842	A-3
	2014	0.6478	0.5061	−0.6725	B-3
	2015	0.5676	0.5203	−4.0463	C-2
	2016	0.6371	0.5334	4.0518	A-1
	2017	0.6597	0.5479	1.0834	A-1
	2018	0.6671	0.6031	0.1009	A-1
Suizhou	2009	0.6557	0.4384	-	-
	2010	0.5495	0.4475	−6.3906	C-2
	2011	0.5542	0.4425	−0.6188	A-3
	2012	0.5625	0.4360	−0.7806	A-3
	2013	0.5575	0.4589	−0.1382	B-3
	2014	0.5010	0.4872	−1.5947	C-2
	2015	0.5136	0.4640	−0.4777	A-3
	2016	0.5654	0.4684	8.8176	A-1
	2017	0.5963	0.4742	3.4725	A-1
	2018	0.6204	0.5118	0.4194	A-1
Enshi	2009	0.5943	0.4186	-	-
	2010	0.6130	0.4284	0.9349	A-1
	2011	0.6582	0.4100	−1.0677	A-3
	2012	0.6602	0.4019	−0.0914	A-3
	2013	0.6860	0.3799	−0.3959	A-3
	2014	0.6483	0.4259	−0.2982	B-3
	2015	0.6548	0.4788	0.0593	A-1
	2016	0.6785	0.5459	0.2076	A-1
	2017	0.7016	0.5488	5.0107	A-1
	2018	0.7132	0.6005	0.1481	A-1
Xiantao	2009	0.6112	0.5171	-	-
	2010	0.6133	0.5373	0.0787	A-1
	2011	0.6225	0.5414	1.7083	A-1
	2012	0.3984	0.5108	8.1675	B-1
	2013	0.5808	0.5193	24.5928	A-1
	2014	0.5293	0.5226	−13.6087	C-2
	2015	0.4972	0.5434	−1.6638	C-2
	2016	0.5884	0.5645	4.5273	A-1
	2017	0.6188	0.5723	3.4903	A-1
	2018	0.5740	0.6438	−0.6487	B-3
Qianjiang	2009	0.6440	0.4917	-	-
	2010	0.6056	0.5000	−2.9050	C-2
	2011	0.5872	0.4915	1.5031	B-1
	2012	0.5936	0.4974	0.7568	A-1
	2013	0.6005	0.4778	−0.2365	A-3
	2014	0.5716	0.5001	−0.9002	B-3
	2015	0.4920	0.4675	2.0321	B-1
	2016	0.5590	0.5238	1.0602	A-1
	2017	0.6288	0.5103	−3.9410	C-2
	2018	0.6148	0.6121	−0.1115	B-3
Tianmen	2009	0.6892	0.4639	-	-
	2010	0.6599	0.4555	1.6035	B-1
	2011	0.6038	0.4569	−20.8449	C-2
	2012	0.6544	0.4938	0.7822	A-1
	2013	0.6012	0.5256	−1.1060	B-3
	2014	0.6591	0.5442	2.2476	A-1
	2015	0.5190	0.5587	−8.6164	C-2
	2016	0.5695	0.5823	2.3474	A-1
	2017	0.6285	0.6002	3.2232	A-1
	2018	0.5785	0.6433	−1.2331	C-2
Shennongjia	2009	0.6143	0.3248	-	-
	2010	0.6077	0.4102	−0.0278	B-3
	2011	0.6196	0.4384	0.2012	A-1
	2012	0.6552	0.4373	−14.9486	C-2
	2013	0.6705	0.4171	−0.3154	A-3
	2014	0.6877	0.4373	0.3384	A-1
	2015	0.6773	0.4710	−0.1360	B-3
	2016	0.7020	0.4724	8.3796	A-1
	2017	0.7349	0.4928	0.7274	A-1
	2018	0.7593	0.5127	0.5559	A-1

## References

[1] Bryan B.A., Gao L., Ye Y., Sun X., Connor J.D., Crossman N.D., Stafford-Smith M., Wu J., He C., Yu D. (2018). China’s response to a national land-system sustainability emergency. Nature.

[2] Lu Y., Zhang Y., Cao X., Wang C., Wang Y., Zhang M., Ferrier R.C., Jenkins A., Yuan J., Bailey M.J. (2019). Forty years of reform and opening up: China’s progress toward a sustainable path. Sci. Adv..

[3] Yue W., Wang T. (2019). Logical Problems on the Evaluation of Resources and Environment Carrying Capacity for Territorial Spatial Planning. China Land Sci..

[4] Pan J. (2012). From Industrial Toward Ecological in China. Science.

[5] Niva V., Cai J., Taka M., Kummu M., Varis O. (2020). China’s sustainable water-energy-food nexus by 2030: Impacts of urbanization on sectoral water demand. J. Clean. Prod..

[6] Xu Y., Xu X., Tang Q. (2016). Human activity intensity of land surface: Concept, methods and application in China. J. Geogr. Sci..

[7] Huang X., Song Y. (2019). Evaluation model of regional resource and environment comprehensive carrying capacity based on the conjugation-wrestling mechanism. J. Nat. Resour..

[8] Zhang F., Wang Y., Ma X., Wang Y., Yang G., Zhu L. (2019). Evaluation of resources and environmental carrying capacity of 36 large cities in China based on a support-pressure coupling mechanism. Sci. Total Environ..

[9] Świąder M., Szewrański S., Kazak J.K. (2020). Environmental Carrying Capacity Assessment—The Policy Instrument and Tool for Sustainable Spatial Management. Front. Environ. Sci..

[10] Feng Z., Yang Y., Yan H., Tao P., Peng L. (2017). A review of resources and environment carrying capacity research since the 20th Century: From theory to practice. Resour. Sci..

[11] Shen L., Shu T., Liao X., Yang N., Ren Y., Zhu M., Cheng G., Wang J. (2020). A new method to evaluate urban resources environment carrying capacity from the load-and-carrier perspective. Resour. Conserv. Recycl..

[12] Fu J., Zang C., Zhang J. (2020). Economic and resource and environmental carrying capacity trade-off analysis in the Haihe River basin in China. J. Clean. Prod..

[13] Su Y., Xue H., Liang H. (2019). An evaluation model for urban comprehensive carrying capacity: An empirical case from harbin city. Int. J. Environ. Res. Public Health.

[14] Cheng X., Long R., Chen H., Li Q. (2019). Coupling coordination degree and spatial dynamic evolution of a regional green competitiveness system—A case study from China. Ecol. Indic..

[15] Xie X., Li X., He W. (2020). A land space development zoning method based on resource–environmental carrying capacity: A case study of henan, China. Int. J. Environ. Res. Public Health.

[16] Zhou J., Chang S., Ma W., Wang D. (2021). An unbalance-based evaluation framework on urban resources and environment carrying capacity. Sustain. Cities Soc..

[17] Cheng J., Zhou K., Chen D., Fan J. (2016). Evaluation and analysis of provincial differences in resources and environment carrying capacity in China. Chinese Geogr. Sci..

[18] Meadows, Donella H., Rome C.O. (1972). The Limits to Growth: A Report for the Club of Rome’s Project on the Predicament of Mankind.

[19] UNESCO, FAO (1985). Carrying Capacity Assessment with a Pilot Study of Kenya: A Resource Accounting Methodology for Exploring National Options for Sustainable Development.

[20] Cohen J.E. (1995). Population growth and earth’s human carrying capacity. Science.

[21] Yang Z., Song J., Cheng D., Xia J., Li Q., Ahamad M.I. (2019). Comprehensive evaluation and scenario simulation for the water resources carrying capacity in Xi’an city, China. J. Environ. Manag..

[22] Su Y., Yu Y. (2020). Dynamic early warning of regional atmospheric environmental carrying capacity. Sci. Total Environ..

[23] Martire S., Castellani V., Sala S. (2015). Carrying capacity assessment of forest resources: Enhancing environmental sustainability in energy production at local scale. Resour. Conserv. Recycl..

[24] Lei X., Qiu G. (2016). Empirical study about the carrying capacity evaluation of regional resources and environment based on entropy-weight TOPSIS model. Acta Sci. Circumstantiae.

[25] Liao S., Wu Y., Wong S.W., Shen L. (2020). Provincial perspective analysis on the coordination between urbanization growth and resource environment carrying capacity (RECC) in China. Sci. Total Environ..

[26] Lane M. (2010). The carrying capacity imperative: Assessing regional carrying capacity methodologies for sustainable land-use planning. Land Use Policy.

[27] Ling X., Zhihong L., Jing D., Dan Y. (2011). Study on Evaluation of Water Ecological Carrying Capacity. Proceedinds of the Biology, Environment and Chemistry.

[28] Liu H. (2012). Comprehensive carrying capacity of the urban agglomeration in the Yangtze River Delta, China. Habitat Int..

[29] Wang D., Shi Y., Wan K. (2020). Integrated evaluation of the carrying capacities of mineral resource-based cities considering synergy between subsystems. Ecol. Indic..

[30] Li K., Jin X., Ma D., Jiang P. (2019). Evaluation of Resource and Environmental Carrying Capacity of China’s Rapid-Urbanization AreasA Case Study of Xinbei District, Changzhou. Land.

[31] Klingebiel S. (1999). Social development and the UN system. Int. Soc. Sci. J..

[32] Grzebyk M., Stec M. (2015). Sustainable Development in EU Countries: Concept and Rating of Levels of Development. Sustain. Dev..

[33] Ng T.H., Lye C.T., Chan K.H., Lim Y.Z., Lim Y.S. (2020). Sustainability in Asia: The Roles of Financial Development in Environmental, Social and Governance (ESG) Performance. Soc. Indic. Res..

[34] Lane M., Dawes L., Grace P. (2015). Scalar considerations in carrying capacity assessment: An Australian example. Popul. Environ..

[35] Luo W., Ren Y., Shen L., Zhu M., Jiang Y., Meng C., Zhang P. (2020). An evolution perspective on the urban land carrying capacity in the urbanization era of China. Sci. Total Environ..

[36] Peng T., Deng H. (2021). Study on the division of main functional regions based on relative carrying capacity of resources: A case study of Guiyang, southwest China. Environ. Dev. Sustain..

[37] Wang X., Zhan W., Wang S. (2020). Uncertain water environment carrying capacity simulation based on the Monte Carlo method–system dynamics model: A case study of Fushun City. Int. J. Environ. Res. Public Health.

[38] Qin G., Li H., Wang X., Ding J. (2016). Research on Water Resources Design Carrying Capacity. Water.

[39] Jia Z., Cai Y., Chen Y., Zeng W. (2018). Regionalization of water environmental carrying capacity for supporting the sustainable water resources management and development in China. Resour. Conserv. Recycl..

[40] Zhou X.-Y., Zheng B., Khu S.-T. (2019). Validation of the hypothesis on carrying capacity limits using the water environment carrying capacity. Sci. Total Environ..

[41] Wang J., Wei X., Guo Q. (2018). A three-dimensional evaluation model for regional carrying capacity of ecological environment to social economic development: Model development and a case study in China. Ecol. Indic..

[42] Chapman E.J., Byron C.J. (2018). The flexible application of carrying capacity in ecology. Glob. Ecol. Conserv..

[43] Wu X., Hu F. (2020). Analysis of ecological carrying capacity using a fuzzy comprehensive evaluation method. Ecol. Indic..

[44] Tapio P. (2005). Towards a theory of decoupling: Degrees of decoupling in the EU and the case of road traffic in Finland between 1970 and 2001. Transp. Policy.

[45] Wang F., Xu H. (2021). Decoupling elasticity of carbon emissions and economic growth of transportation industry in Zhejiang province and its influencing factors. J. Environ. Prot. Ecol..

[46] Wu X., Wang L., Zheng H. (2019). A network effect on the decoupling of industrial waste gas emissions and industrial added value: A case study of China. J. Clean. Prod..

[47] Mao H.Y., Yu D.L. (2001). A study on the quantitative research of regional carrying capacity. Adv. Earth Sci..

[48] Peng T., Deng H. (2020). Comprehensive evaluation on water resource carrying capacity based on DPESBR framework: A case study in Guiyang, southwest China. J. Clean. Prod..

[49] Chen C.H., Wu R.S., Liaw S.L., Sue W.R., Chiou I.J. (2000). A study of water-land environment carrying capacity for a river basin. Water Sci. Technol..

[50] Liu R.Z., Borthwick A.G.L. (2011). Measurement and assessment of carrying capacity of the environment in Ningbo, China. J. Environ. Manag..

[51] Jung C., Kim C., Kim S., Suh K. (2018). Analysis of environmental carrying capacity with emergy perspective of Jeju Island. Sustainability.

[52] Zhou Y., Zhou J. (2017). Urban atmospheric environmental capacity and atmospheric environmental carrying capacity constrained by GDP–PM_2.5_. Ecol. Indic..

[53] OECD (1982). The OECD List of Social Indicators.

[54] UNDP (2020). HumanDevelopmentReports.

[55] Tang J. (1999). Comparison of Comprehensive Indexes of Social Development. Stat. Decis..

[56] China National Bureau of Statistics (1993). Comprehensive Evaluation Scheme of Social Development Level.

[57] Friedman J.H., Tukey J.W. (1974). A Projection Pursuit Algorithm for Exploratory Data Analysis. IEEE Trans. Comput..

[58] Zhu Z., Chen Z., Chen X., He P. (2016). Approach for evaluating inundation risks in urban drainage systems. Sci. Total Environ..

[59] Tang Q., Wang J., Jing Z. (2021). Tempo-spatial changes of ecological vulnerability in resource-based urban based on genetic projection pursuit model. Ecol. Indic..

[60] Peng B., Zhang X., Elahi E., Wan A. (2021). Evolution of spatial–temporal characteristics and financial development as an influencing factor of green ecology. Environ. Dev. Sustain..

[61] State Council of China: Guiding Opinions of the State Council on Promoting the Development of the Yangtze River Economic Belt Based on the Golden Waterway. http://www.gov.cn/zhengce/content/2014-09/25/content_9092.htm.

